# Ectopic Ureter: Spectrum of Magnetic Resonance Imaging Findings

**DOI:** 10.7759/cureus.58977

**Published:** 2024-04-25

**Authors:** Kavita Rani, Pragya Surolia, Usha Jaipal, Naima Mannan

**Affiliations:** 1 Radiodiagnosis, Sawai Man Singh (SMS) Medical College, Jaipur, IND

**Keywords:** vagina, seminal vesicle, duplex kidney, ectopic ureter, zinner syndrome

## Abstract

Objective: This study aims to describe the MRI findings of six patients with ectopic ureters in a tertiary care institute.

Methods: A retrospective analysis was conducted on six patients presenting to the Department of Radiodiagnosis at Sawai Man Singh (SMS) Hospital, Jaipur, India, with ectopic ureters. Data were collected from the 3 Tesla (3T) Philips MRI scanner (Koninklijke Philips N.V., Amsterdam, Netherlands) from 2021 to 2023.

Results: The mean age was 21.6 years, with an equal male-to-female ratio (1:1). Most patients presented with urinary incontinence (50%, n = 3), followed by abdominal pain. Only 16.6% (n = 1) were associated with a duplex collecting system. In males, the most common site was the seminal vesicle, observed in 66.6% (n = 2), followed by the urethra in 33.4% (n = 1) of cases. One patient with an ectopic opening into the seminal vesicle had Zinner syndrome. In females, the ectopic site was found to be the vagina in all three patients.

Conclusion: Ectopic ureter is a rare anomaly of the urinary system, often associated with other urinary system anomalies and a few syndromes. Clinical presentations range from the patient being asymptomatic to renal failure; therefore, a high index of suspicion and appropriate imaging are necessary for early diagnosis and timely treatment.

## Introduction

An ectopic ureter is defined as the insertion of the ureter in an area other than the trigonal region due to the caudal migration of the ureteric bud during its insertion into the urinary bladder. Women are affected twice as often as men, and the majority of cases are asymptomatic [1, 2]. In females, the most common sites for ectopic ureters are the bladder neck/upper urethra, followed by the vestibule, vagina, cervix, and uterus. In males, ectopic ureters typically open into the lower part of the urinary bladder, followed by the posterior urethra, seminal vesicle, vas deferens, and ejaculatory duct. Approximately 30% of cases involve a duplex collecting system with an ectopic ureter, while the remainder have a single system with an ectopic ureter, as discussed in the study by Choudhury et al. [3]. Bilateral single-system ectopic ureters are even rarer [4]. Symptoms are often related to the site of the ureteral opening. Diagnosis may be delayed in asymptomatic patients; females typically present with urinary incontinence, while males may present with recurrent infections. In this article, we have analyzed six patients with ectopic ureters.

## Materials and methods

This retrospective study was conducted after obtaining ethical committee approval and the necessary informed consent. Six patients with ectopic ureters were selected for this study by analyzing data obtained from the 3 Tesla (3T) Philips MRI scanner (Koninklijke Philips N.V., Amsterdam, Netherlands) from 2021 to 2023 at the radiology department of Sawai Man Singh (SMS) Hospital, Jaipur, India. All patients had undergone magnetic resonance (MR) urography with MRI sequences as shown in Table 1. Three-dimensional (3D) reformation of the urinary system was performed to visualize the complete course of the ureter and its opening. Contrast was administered to the patients as needed.

**Table 1:** MRI sequences for MR urography SPAIR: spectral attenuated inversion recovery; DWI: diffusion-weighted image; MIP: maximum intensity projection; 3D: three-dimensional

Sequences	Plane
T2	Axial, coronal
T2 SPAIR	Axial, coronal
T1	Axial
DWI	Axial
3D, MIP	Coronal
Post-contrast T1	Axial, coronal, and sagittal

## Results

A total of six patients were studied, ranging in age from 10 years to 48 years, with a mean age of 21.6 years. The majority of patients were adolescents (83.4%, n = 5), with only one adult patient (16.6%). The male-to-female ratio was equal (1:1).

All female patients (50%) were symptomatic, presenting with urinary incontinence. Lower abdominal pain was the next most common symptom, observed in 33.3% of patients (n = 2). Only one patient (16.7%) was asymptomatic.

Only 16.6% of cases (n = 1) were associated with a duplex collecting system, in which the upper moiety ureter was fused with the lower moiety ureter, both opening together into the left lateral wall of the vagina. The remaining 83.4% of cases (n = 5) had a single collecting system. Only 16.6% of cases (n = 1) had a normal ipsilateral kidney with a duplex collecting system, while 50% of cases (n = 3) had atrophic ipsilateral kidneys, and 33.4% (n = 2) had an absent ipsilateral kidney.

In males, the most common site was the seminal vesicle, observed in 66.6% of cases (n = 2), followed by the urethra in 33.4% of cases (n = 1). One patient with an ectopic opening into the seminal vesicle had Zinner syndrome.

In females, the most common ectopic site was the vagina in all three cases. Hydroureter was observed in 66.6% of cases (n = 4) without hydronephrosis, while the remaining 33.4% of cases (n = 2) did not show dilatation of the involved ureter. Clinical presentation and MRI findings are summarized in Table 2.

**Table 2:** Summary of the six patients having an ectopic ureter including their clinical and MRI findings

S. No.	Age (years)/Sex	Symptoms	Collecting system	Side	Ipsilateral kidney	Ectopic site
1.	48/Male	Lower abdominal pain	Single	Left	Atrophic	Seminal vesicle
2.	21/Female	Incontinence	Single	Right	Absent	Vagina
3.	10/Female	Incontinence	Duplicated	Left	Normal	Vagina
4.	13/Male	Asymptomatic	Single	Left	Atrophic	Urethra
5.	18/Male	Abdominal pain	Single	Left	Absent	Seminal vesicle
6.	20/Female	Incontinence	Single	Left	Atrophic	Vagina

## Discussion

An ectopic ureter is a rare condition, and diagnosing it can be delayed in asymptomatic patients. A high level of suspicion is necessary in asymptomatic individuals to confirm the diagnosis. The incidence of ectopic ureter is rare, approximately 0.05%-0.025% [5]. Various imaging modalities are available to identify ectopic ureter insertion, including ultrasonography, intravenous pyelography (IVP), micturating cystourethrography (MCU), CT urography, and MRI. Magnetic resonance imaging and MR urography are particularly effective in diagnosing cases where other modalities are inconclusive [6-8]. An MRI has greater soft tissue contrast and high resolution, which makes it superior to other modalities, and imaging in multiple planes can also be taken. No radiation exposure in MRI makes it suitable for all patients, especially children and females.

We have reported six cases of ectopic ureter, describing their MRI findings. Women are typically affected twice as often as men, and most cases are asymptomatic [1, 2]. However, in our study, the male-to-female ratio was equal (1:1). Only one patient was asymptomatic, while two had lower abdominal pain. All three female patients presented with urinary incontinence, which is a common complication of ectopic ureter.

The ectopic ureter may be associated with various anomalies, such as duplex kidneys, duplication of the ureter, ureterocele, and syndromic associations like VACTERL (which stands for vertebral defects, anal atresia, cardiac defects, tracheoesophageal fistulas, renal anomalies, and limb abnormalities) syndrome and Zinner syndrome. Duplex collecting systems with an ectopic ureter occur in approximately 30% of cases, with the remainder having a single system with an ectopic ureter, as shown in the study by Choudhury et al. [3]. In our study, 16.6% of patients had a duplex collecting system, and 83.4% had a single collecting system. Also, 83.4% of cases showed isolated left system involvement, while 16.6% had isolated right system involvement; no cases of bilateral involvement were observed in our study.

**Figure 1:**
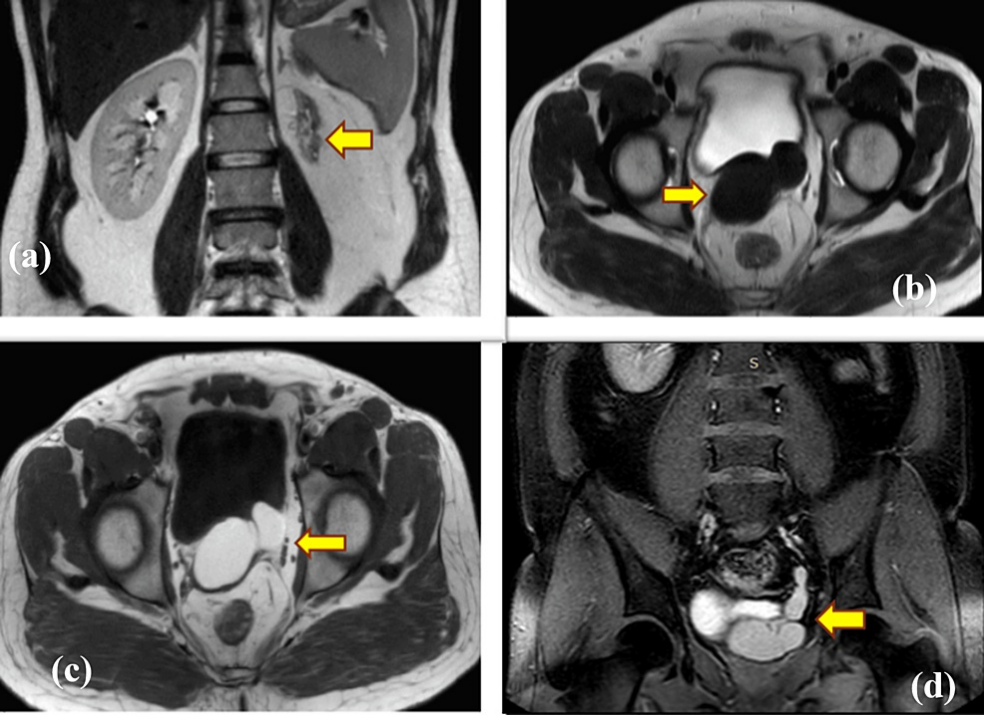
(a) Coronal T2-weighted image shows an atrophic left kidney (yellow arrow), while the right kidney appears normal. (b) The axial T2-weighted image shows a hypointense cystically dilated seminal vesicle, which appears hyperintense on the axial T1-weighted image (c), suggesting a high proteinaceous or hemorrhagic content. (d) The coronary post-contrast T1-weighted image demonstrates the communication of the cystically dilated seminal vesicle with the left ureter, indicating ectopic insertion of the ureter into the seminal vesicle.

In our study, we observed one case of Zinner syndrome in an 18-year-old individual. This patient showed left renal agenesis, ejaculatory duct obstruction, seminal vesicle cysts, as well as ectopic insertion of the remnant ureter into the ipsilateral seminal vesicle. Zinner syndrome, first described in 1914 by Zinner, represents a rare congenital anomaly involving the seminal vesicles and the ipsilateral upper urinary tract [9]. The classic triad of seminal vesicle cysts, ipsilateral renal agenesis, and ipsilateral ejaculatory duct obstruction defines Zinner syndrome. However, in addition to this triad, patients may also present with other genitourinary disorders such as ureterocele, hypospadias, and disorders of the testicular, epididymal, or adrenal glands [9]. Zinner syndrome results from the atresia of both the ejaculatory duct and the ureteric bud due to the maldevelopment of the distal part of the mesonephric duct before the seventh week of gestation [10]. Ectopic insertion of the remnant ureter into the seminal vesicles is exceedingly rare. A similar case has been reported by Palacios et al. [11], where they discussed the ectopic insertion of the left ureteric remnant in a patient with Zinner syndrome into the seminal vesicle. We also observed another patient with ectopic ureter insertion into a cystically dilated seminal vesicle, accompanied by an atrophic ipsilateral kidney (Figures 1a-1d).

In males, ectopic insertion of the ureter into the urethra was observed in approximately 50% of cases. In our study, 33.3% of patients had ectopic urethral insertion of the ureter. Deng et al. [12] have reported a case of ectopic insertion of a duplicated ureter into the prostatic urethra.

**Figure 2:**
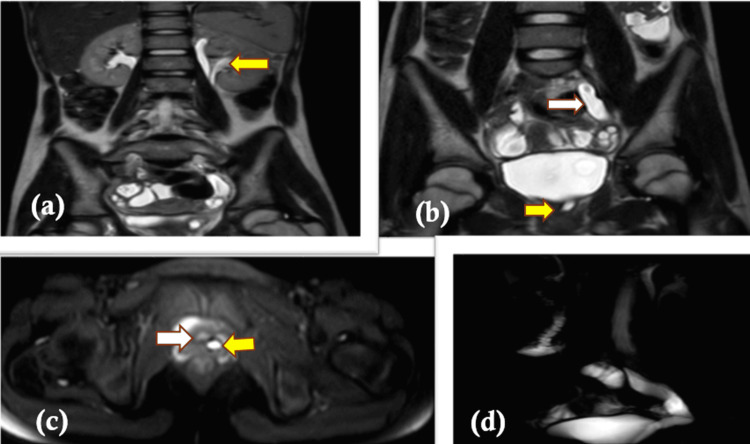
(a) The coronal T2-weighted image shows a duplex left collecting system (yellow arrow). The right kidney appears normal. (b) The coronal T2-weighted image shows the fusion of the left upper and lower moiety ureters (white arrow), both opening together into the left lateral wall of the vagina (yellow arrow). (c) The axial T2 SPAIR image shows the opening of the ureter (yellow arrow) into the left lateral wall of the vagina (white arrow). (d) A 3D-reformatted image delineating the urinary tract SPAIR: spectral attenuated inversion recovery; 3D: three-dimensional

In females, the vagina represents about 25% of sites of ectopic insertion of the ureter after upper urethra insertion (33%) and the vestibule (33%) [13]. In our study, all three female patients had ectopic insertion of the ureter into the vagina, and one of them had a duplex collecting system (Figures 2a-2d). Duplication of the ureter, together with ectopic ureteric insertion, is also a very rare entity. Singh et al. [14] have also described a case of a duplex collecting system with the opening of an ectopic ureter into the vagina.

The complications of ectopic ureter include recurrent urinary tract infections, urinary incontinence, hydronephrosis, and even renal failure. In our study, 66.6% of cases had hydroureters without associated hydronephrosis; 50% of patients presented with urinary incontinence, and 33.3% had non-functioning atrophic kidneys. Sometimes, patients may present with surgical emergencies such as acute urinary retention, a urinary tract infection leading to sepsis, or a ruptured ureterocele. There are case reports of misplaced urinary catheters into ectopic ureters in previously undiagnosed duplex ureters [15]. Hence, timely diagnosis is essential to planning treatment accurately before complications develop.

Various surgical methods are available to prevent recurrent episodes of infection, preserve renal function, and restore continence [16]. These include reimplantation with bladder neck reconstruction, uretero-renal or uretero-ureteric anastomosis, nephroureterectomy, and heminephrectomy, depending on the insertion site of the ectopic ureter.

There are a few limitations to this study, one of which is the small sample size due to the rarity of the condition. Additionally, since it was a retrospective study, proper follow-up of patients until the final diagnosis and treatment provided could not be assessed.

## Conclusions

An ectopic ureter is a rare anomaly of the urinary system, occasionally associated with other urinary system anomalies and a few syndromes. Clinical presentations range from asymptomatic cases to renal failure. Magnetic resonance urography is a valuable modality for evaluating urinary system anomalies, given its high spatial resolution and 3D reformation techniques. Various surgical methods are available to treat such conditions. Having a high index of suspicion and appropriate imaging for early diagnosis are crucial to accurately planning treatment before complications develop.
